# Supplementary material for: Glioblastoma disrupts cortical network activity at multiple spatial and temporal scales
Source: Nat Commun. 2024 May 27;15:4503. doi: 10.1038/s41467-024-48757-5 (PMC11130179; doi:10.1038/s41467-024-48757-5)
Supplement: Supplementary file 1 — Supplementary Information [file 41467_2024_48757_MOESM1_ESM.pdf]

Glioblastoma disrupts cortical network activity at multiple spatial and temporal scales

Supplementary methods and results

Chronic calcium activity analysis at fast timescales and mesoscopic spatial scales enables analysis of the dynamical range of influence inside and beyond the tumor margin in 3xCR vs GPC6 tumor animals. Here we show a flow chart style schema of calcium widefield analysis in relation to tumor distance, and how we tested for significance between distance and activity metrics (supplementary fig 1). In each recording, we acquired functional calcium or glutamate movies, and snapshots of the tumor (in a separate optical channel). Calcium movies were spatially and temporally downsampled, and periods of active whisking, running and visual stimulation were excluded from further analysis (A). The tumor outline was computed from the other optical channel, and all pixels were assigned corresponding distance bands in 0.75mm-increments from the tumor edge (B). For each binned pixel from the calcium movie, a ΔF/F trace was computed, and after detrending and denoising, individual transients were identified. Mean ΔF/F, event rate, amplitude, area (or duration), and rhythmicity were computed (C). Activity metrics were further analyzed as a function of distance in 2 ways: we computed the p-values of linear fits, and tested for statistical significance by creating a bootstrapped null distribution by circularly shuffling the ΔF/F traces 500,000 times for each pixel (D, see also supplementary figure 5), or we calculated the average metric values for each distance band (E) and compared the ratios between near (<0.75mm) and far (>3mm) from the edge depending on tumor expansion rate and tumor genotype (see figure 5C/D in the main results section).

For cellular resolution images of tumor cells and neuronal calcium activity, we employed an analysis pipeline partially based on previously published literature (see methods section), and extended it to extract information relevant to our analysis. Briefly, we started by identifying neurons using a modified CNMF algorithm. An example of two identified neurons in a motion-corrected spiral scan FOV is outlined in supplementary fig 2A). Underneath we present one minute of the raw calcium traces, intermediately detrended and normalized ΔF/F traces, and lastly the deconvolved "dΔF/F" traces for both neurons. For example neuron 2 we show a detailed view of 10 seconds of dΔF/F activity to visualize how separate calcium events were identified. Next, we extract from the identified events 4 distinct metrics that produce one mean value for each cell in each recording, and these values were ultimately pooled into distributions for statistical comparisons: 1) average dΔF/F is the mean of each cell's dΔF/F values across all samples in a

recording. Here the semilogarithmic histograms show the binned count of these values for a 20-min recording. 2) Event rate: instances of identified events were counted and divided by the number of seconds in each recording. The histograms depict events/sec in 10-sec bins of the same recording used in 1). 3) Event amplitude: To characterize the instantaneous firing (or “burst”) behavior of individual neurons, we calculate the mean event amplitude for each cell. If two cells had the same mean dΔF/F value, then the one with the higher mean amplitude would presumably have a more burst-like firing pattern, and the one with a lower amplitude would be producing more single spikes. The histogram was constructed analogously to the ones in 1). 4) Using the dΔF/F in bins of 100 msec, we first determined Pearson’s pair-wise correlation coefficients for all combinations of individual neurons. Then we apply an algorithm that identifies groups of neurons more likely to be coactive than expected by chance (see methods for details), and color-code them for visualization. In this recording, the two example neurons above were part of the same cluster. The algorithm then computes a clustering coefficient for each neuron.

We performed a bioinformatics analysis of GPC6 tumor brain genes, and show significantly upregulated genes in supplementary fig 3. These genes of interest are organized by different ontology databases. Only significantly modulated genes are shown (dashed red line represents the $p = 0.05$ cutoff).

The human RNA data analysis was performed through the GEPIA online tool (Tang et al, Nuclei Acids Res 2017, PMID: 28407145) containing the RNA-Seq datasets from the UCSC Xena project (http://xena.ucsc.edu). To probe the source of GPC6 further, we utilized publically available studies (Broad Institute, https://portals.broadinstitute.org/single_cell/study/SCP393/single-cell-rna-seq-of-adult-and-pediatric-glioblastoma, Neftel et al, Cell 2019). In supplementary figure 4, we can clearly see that the detectable expression of GPC6 is restricted to the tumor cells. Additionally, there are clearly clusters that express GPC6 in both adult and pediatric samples of high grade glioma.

To address the differences between subtypes, we performed more detailed survival analysis, isolating different glioma subtypes (supplementary Fig 4): We show Kaplan-Meier survival curves differentiating across different glioma subtypes, with data derived from TCGA. Curves were generated using the GlioVis tool (REF: Bowman et al, Neuro-Oncol 2017, PMID: 28031383). Each curve compares the survival of the upper and lower halves of GPC6 expression within patient tumors.

Using our chronic cranial window technique, we were able to image the same animals for many weeks without significant loss of optical clarity and fluorescence intensity. To demonstrate the high degree of clarity over long periods of time, we show widefield images of a GPC6 tumor brain (supplementary Fig 6A), labeled with GFP (red light excitation, green emission filter), and a calcium indicator (jrGeCO1a, green light excitation, red emission). We show the window at 10 different time points between P62 and P141 in the same animal. Similarly, the window of an example 3xCR mouse (tumor: BFP, blue emission filter, 400nm excitation; calcium: thy1-GCaMP6s, green emission filter, blue excitation light) remained clear from P56-99 (supplementary Fig 6B). For visualization, brightness scales were adjusted by normalizing with respect to background signal generated from the lumen of the large pial surface veins.

In order to visualize spatial relationships between calcium or glutamate baseline fluorescence with tumor size, over time, we show dual-color aligned reference images for the 3 example animals (from fig 4 in the main text), in supplementary fig 7 (A: 3xCr tumor, calcium reporter; B: 3xCr tumor, glutamate reporter; C: GPC6 tumor, calcium indicator.

We calculated distance bands in 0.75 mm increments, as shown in supplementary figure 8A/B for 3xCR and GPC6, respectively, averaging the ΔF/F signal concentrically from the tumor border (outlined in white lines). In these examples, we determined significant differences by comparing event rates to a R^2 goodness of fit value analysis. In both 3xCr and GPC6 tumor brains, there was a significantly higher event rate proximal to the tumor (p-values .02 and 2e-6, respectively) during the fast expansion phase. Interestingly, the R^2 of event rate as a linear function of distance from the tumor during slow phases was not significant in either tumor model. This suggests that distance is only a relevant factor underlying cross-talk during the fast expansion phase.

Subsequently, we applied this analysis to the activity metrics (amplitude, area and event rate ("ΔF/min", and "rhythmicity" are shown in supplementary figure 8): In 3xCR animals, linear fit p-values (distance from tumor edge vs. activity metrics) were significantly different between slow and fast tumor expansion periods for event amplitude and area, but not event rate (supplementary fig 8C). In some recordings this was also seen for overall DF/F activity and rhythmicity (mean p-values: 0.57 ± 0.08 sem and 0.22 ± 0.09 sem slow/fast respectively, p = 0.06, KW/mc test; and mean p-values (slow/fast): 0.42 ± 0.06 sem / 0.36 ± 0.1 sem, p = 0.4 KW/mc test), percentages of significant recordings (0% vs 15%, p = 0.07, χ^2 test). Rhythmicity in significant recordings, taken when tumor expansion was fast, dropped by 83% at a distance of 3.75 mm, on average (supplementary fig 8C). For each metric, boxplots contain the corresponding R2 p-values from 20 recordings acquired during slow tumor growth (left), and from 13 recordings during fast growth

(right). The horizontal line corresponds to the median, the vertical extent of the box equals the interquartile range (25th to 75th percentile), the whiskers extend to the most extreme data points not considered outliers, and the outliers are plotted individually using the '+' marker symbol. Notches display the variability of the median between samples, and boxes whose notches do not overlap have different medians (at $\alpha = 0.05$). Pie charts show the percentage of significant recordings under both growth conditions for each metric. The shaded errorbar plots (mean and sem) underneath contain metric data from the corresponding significant recordings, normalized by the first value (corresponding to <0.75 mm distance from the tumor edge) of each recording. The x-axes of these plots extend out to variable maximal values, depending on the tumor coverage of each FOV constraining space for the distance bands.

To analyze whether the number of significant recordings differed between the two expansion rate conditions, we performed a χ^2 test between the percentage of recordings that had a significant linear relationship between distance and activity metrics (pie charts). In 3xCR animals, calcium event sizes were significantly different depending on tumor distance: mean amplitudes were more likely to drop off with distance when tumors spread fast rather than slow (mean p-values: 0.22 ± 0.05 sem and 0.0273 ± 0.01 sem respectively, p = 0.001, KW/mc test). We determined that 31% of recordings in fast expansion states were significant vs. 5% during slow periods (p = 0.04, χ^2 test). Mean amplitudes in slow period recordings were 70% lower at 3mm from the edge, and 80% lower at 3.75mm under fast spreading conditions. Similarly, calcium event areas had a stronger association with distance under fast than slow conditions (mean p-values: 0.047 ± 0.05 sem vs 0.28 ± 0.05 sem respectively, p = 0.002, WR test), and 46% of recordings were significant vs 0% (p = 8e-4, χ^2 test). Mean area values were on average 83% lower at a distance of 4.5 mm than <0.75mm from the tumor margin. However, event rates were, in most recordings, not significantly associated with distance from the tumor, and there was no significant difference between slow and fast expansion periods (mean p-values: 0.4 ± 0.06 sem vs. 0.34 ± 0.105 sem, p = 0.32 WR test). While 2 recordings during fast expansion showed significance, there were none during slow periods, but this difference was not significant (p=0.07, χ^2 test). When significantly modulated, event rate was on average 83% lower at a distance of 3.75 mm from the tumor edge.

Whereas these results from 3xCR tumor animals indicate that overall calcium events were progressively diminished at increasing distances from the tumor, GPC6 peritumoral activity generally did not follow the same activity-distance relationships (supplementary fig 8D). There were no recordings with a significant correlation of distance and amplitude or area (empty pie

charts for ΔF/min, rhythmicity), and goodness-of-fit p-values were never different significantly different between recordings from slow and fast growing tumor epochs (amplitude: 0.28 ± 0.07 sem and 0.29 ± 0.11 sem, p = 0.65 WR test; area: 0.27 ± 0.07 sem and 0.3 ± 0.13 sem, p = 0.8, WR test). However, the event rate was significantly modulated as a function of distance in two GPC6 recordings, one while the tumor was growing slowly, and one fast. In accordance with these low numbers, there was no significant difference between the two conditions (mean p-values: 0.38 ± 0.06 sem and 0.34 ± 0.12 sem, p = 0.7 KW/mc test), nor between the percentage of significant recordings (5% vs 12%, p = 0.49, χ^2 test). In the significant slow period recording, mean event rates were 16% lower at a distance of 3 mm, and 42% lower at 6 mm. The maximum distance in the recording taken during a period of fast tumor expansion was 3 mm.

We examined whether intramarginal somatic activity differed in similar ways from cortical activity recorded in non-tumor control animals. We found that in 3xCR tumor animals, mean focal dΔF/F (i.e. AP production) measured inside the tumor margin was unchanged compared to controls. However, the mean event rate was elevated by 52% (p = 1e-13, KW/mc test), and amplitude by 17.7% (p = 2e-3, KW/mc test, supplementary figure 9A, left). Neuronal calcium event rates inside GPC6 tumor margins were 36% higher than controls (p = 4e-9, KW/mc test), and amplitudes 15% higher (p = 6e-5, KW/mc test). However, there was also a 26% reduction of overall calcium activity compared to controls (p = 3e-3, KW/mc test, supplementary figure 9A, right).

Supplementary figure 1 (supplement to figure 1)

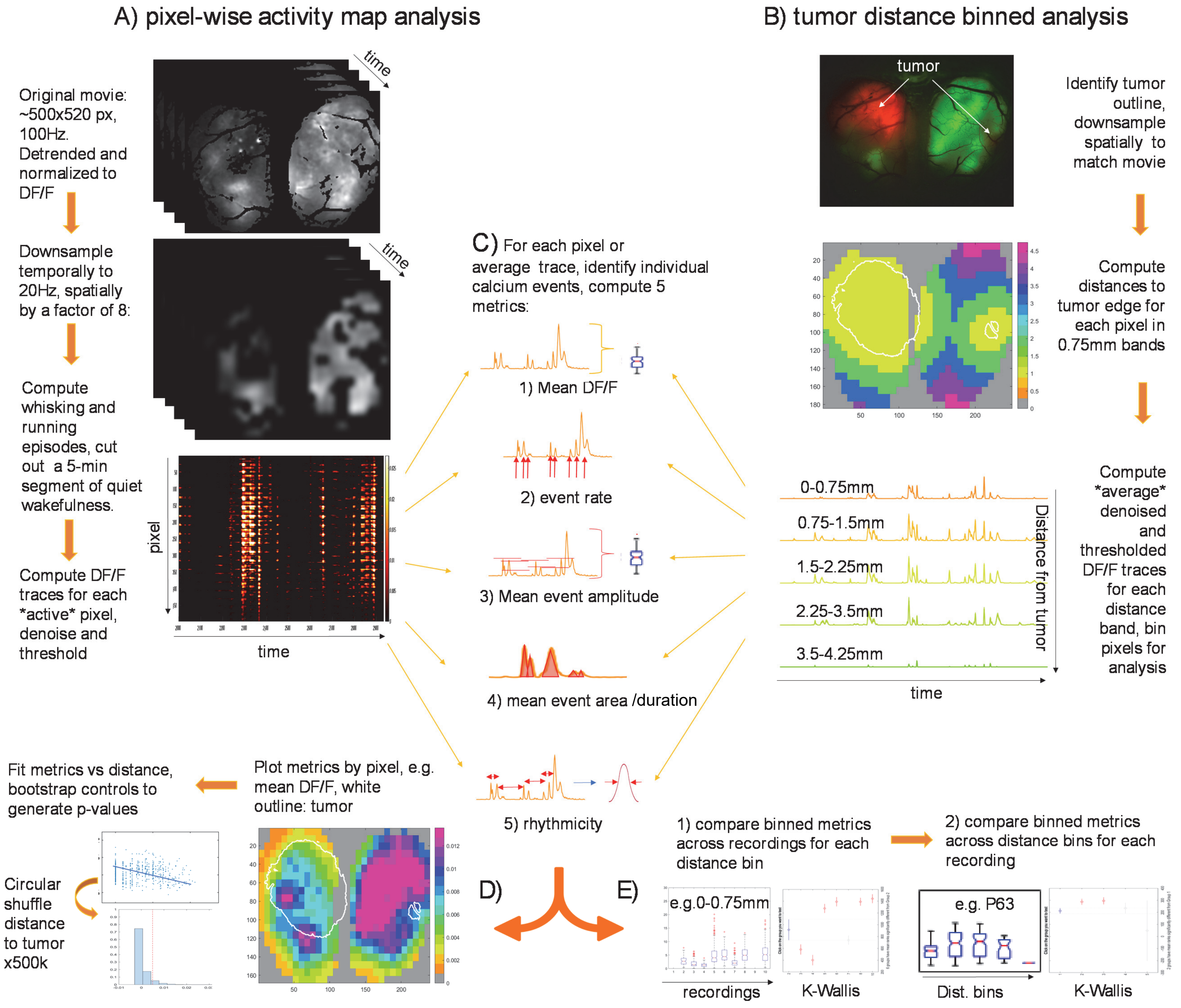

Supplementary. Fig. 1 (supplement to Figure 1): Chronic calcium activity analysis at fast timescales and mesoscopic spatial scales enables analysis of the dynamical range of influence inside and beyond the tumor margin in 3xCR vs GPC6 tumor animals

A) 1-p widefield images are recorded at ~500x520 pixel resolution and 100Hz sampling rate. Images are downsampled temporally to 20Hz and spatially by a factor of 8, resulting in ~0.24mm pixel resolution. After computing active whisking and running periods, 300 sec of quiet wakeful activity is selected. For each pixel, a corresponding dΔF/F signal trace is computed, detrended, and thresholded at 3 SD above mean baseline noise level.

B) Using a snapshot image taken with a green/red filter (for RFP labeled tumor) or 400nm/blue filter (for BFP labeled tumor), the tumor margin is computed for each recording at different time points. This image is aligned and spatially downsampled as in (A), and pixels are assigned to distance bands of 0.75 mm width. Average ΔF/F traces are calculated for visualization, and pixels are binned into the respective 0.75 mm distance bands.

C) Each ΔF/F trace is processed to extract the following metrics: I) mean ΔF/F over the duration of each trace, ii) calcium transient event rate sec^{-1}), iii) mean event amplitude for each trace, iv) mean area under the curve of identified calcium events, and v) rhythmicity, i.e. the inverse of the width at half maximum amplitude for the distribution of inter-event intervals.

D) To visualize changes in activation patterns over time, values for the computed metrics are plotted pixel by pixel with the outline of the tumor overlaid in white. To determine a possible relationship between the distance to the tumor edge and the neuronal activity metrics, the linear fit R^2 is computed. Next, distances to the tumor edge are scrambled via circular shuffling 500k times to create a bootstrapped null distribution and a p-value for the significance of the correlation.

E) Next, metric distributions for pixels binned into 0.75 mm-distance bands are computed, and compared, first across time for each distance bin, and then across distance for each time point (Kruskal-Wallis test with correction for multiple comparisons).

Supplementary figure 2 (supplement to figure 1)

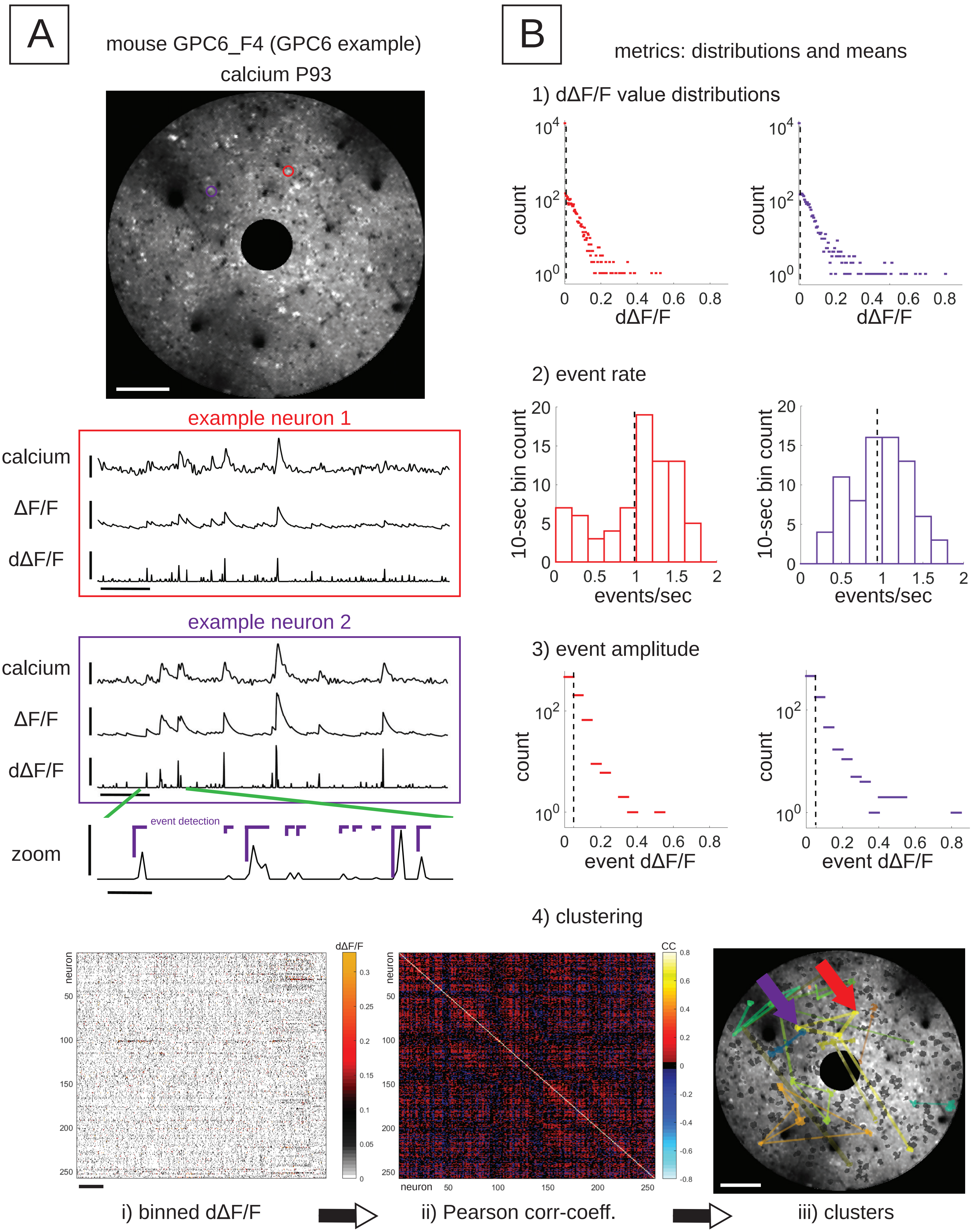

Supplementary Fig. 2 (supplement to Figure 1): 2-photon calcium imaging data analysis pipeline: processing raw calcium data and extracting metrics and coactive clusters

A) Top: example FOV snapshot from a GPC6 tumor mouse recorded at P93, neurons labeled with AAV-jrGeco1a. Scale bar = 100 um. Purple and red circles: example neurons whose calcium activity traces are plotted below. Bottom: raw calcium traces, preprocessed ΔF/F, and deconvolved ΔF/F from the two example neurons highlighted in A). Horizontal scale bar = 10 sec. Vertical scale bars (from top to bottom, for both example neurons: 10,000 A.U. (digitized at 12 bit resolution and upsampled to 16-bit), 100% ΔF/F, 1 A.U. (equivalent to spike probability). Horizontal scale bar for the zoomed in portion of the example neuron2 deconvolved trace: 1 sec. The purple bars represent the identified event durations (horizontal) and amplitudes (vertical).

B) Extraction of activity metrics from the two example neurons in A): 1) mean dΔF/F: semilogarithmic histogram of deconvolved dΔF/F values across a 20-min recording. 2) events/sec: histogram of event rates binned in 10-sec increments, from the same 20-min recording. 3) Event amplitude: semilogarithmic histogram of event amplitude values across the same 20-min segment. Dashed vertical lines (in all 6 plots) = mean values for each metric. 4) i): deconvolved ΔF/F traces of ca. 250 neurons, binned in 100-msec increments. Horizontal scale bar = 10 sec. ii): matrix of pair-wise Pearson correlation coefficients for all possible pairs of the 250 neurons. iii): visualization of significantly coactive clusters of neurons. Arrows: example neurons 1 and 2 were part of the cluster of neurons connected by yellow lines.

Supplementary figure 3 (supplement to figure 2)

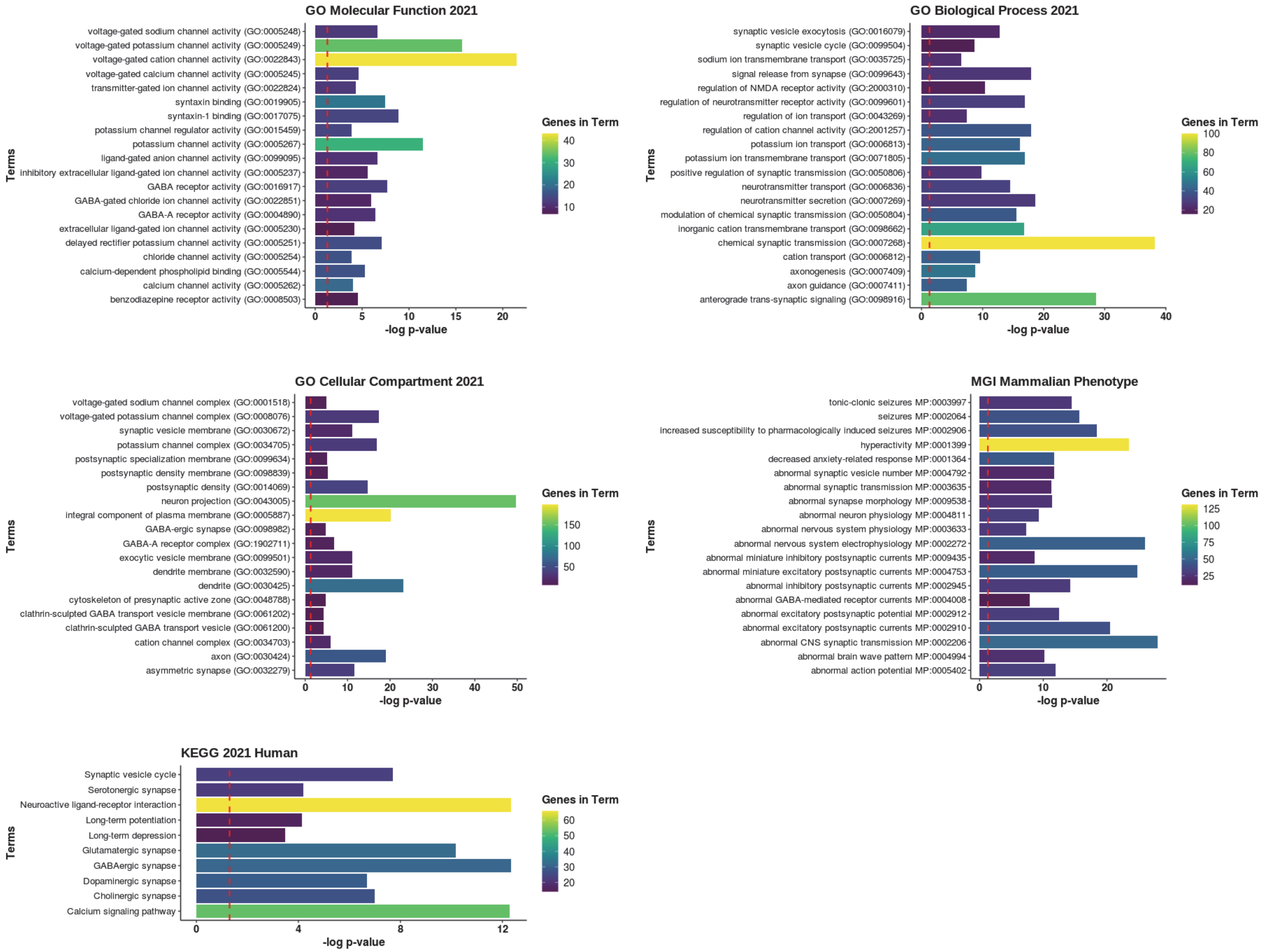

Supplementary Fig. 3 (supplement to Figure 2): Bioinformatics analysis of differentially upregulated genes in GPC6 tumor brains.

Bar graphs demonstrate of different significance values of various terms from different ontology databases. Significance values were calculated as the –log(p-value). Colors of bars indicate the number of genes in each term that were found. Dotted red line set at p = .05 threshold.

Supplementary figure 4 (supplement to figure 2)

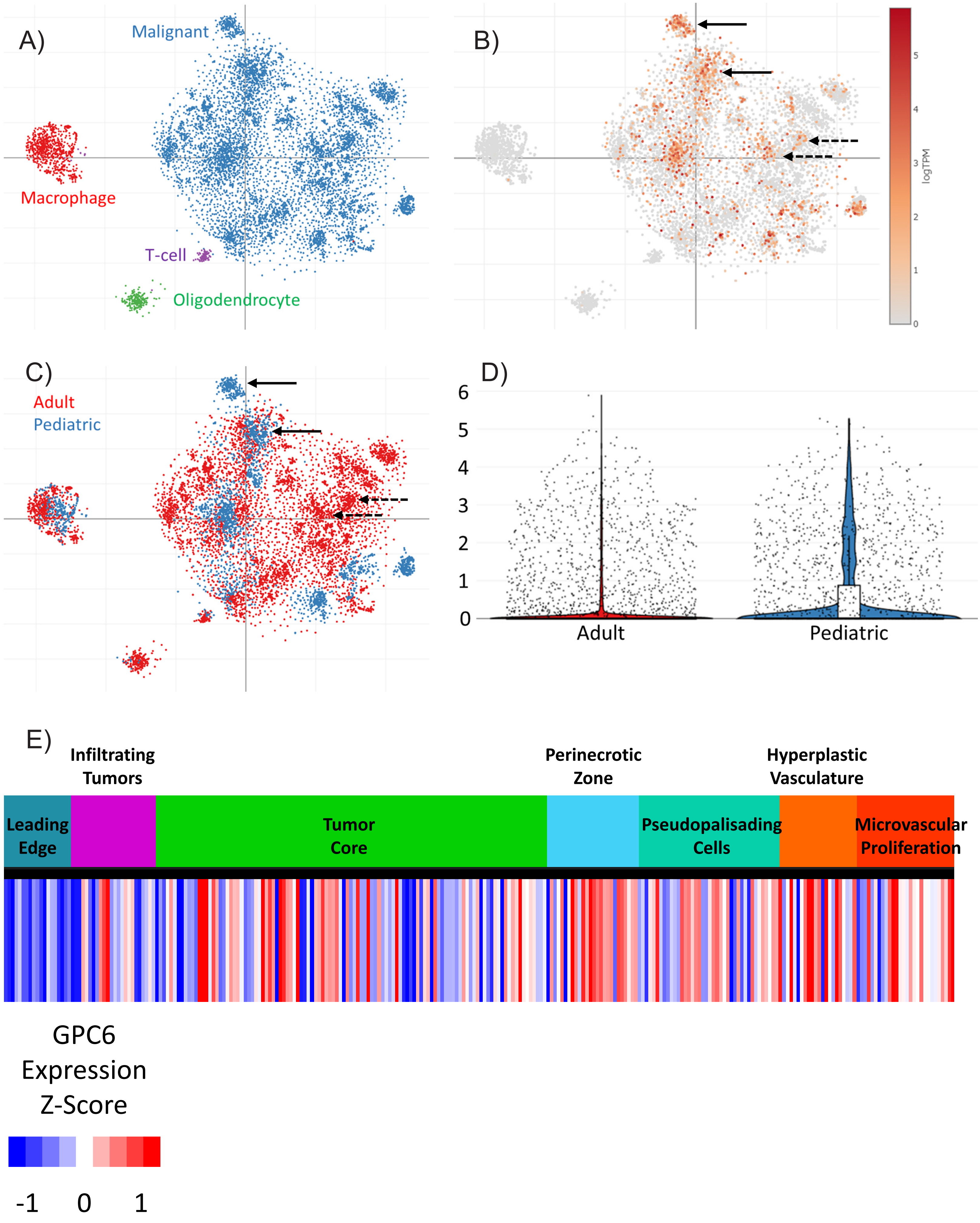

Supplementary Fig. 4 (supplement to Figure 2): GPC6 expression is restricted to tumor cells.

A) Single cell RNA-Seq analysis of adult and pediatric GBM from publically available dataset (REF: Neftel et al, Cell 2019, PMID: 31327527) annotated different cell types. Cells are mapped by tSNE clustering. Horizontal axis is tSNE1, vertical axis is tSNE2. B) Expression plot of GPC6 demonstrates expression is restricted to tumor cells only. C) Plot annotating cells from adult (red) and pediatric (blue) samples. Arrows indicate GPC6 expression (from B) is found in both adult (dashed arrows) and pediatric (solid arrows) samples. D) Comparison of expression between adult and pediatric samples. Y-axis values represent log TPM (transcript per million). E) Analysis of spatial transcriptomic data from publically available dataset (REF: Puchalski et al, Science 2018, PMID: 29748285) demonstrates GPC6 expression is restricted to within the tumor or the invasive infiltrating cells (purple).

Supplementary figure 5 (supplement to figure 2)

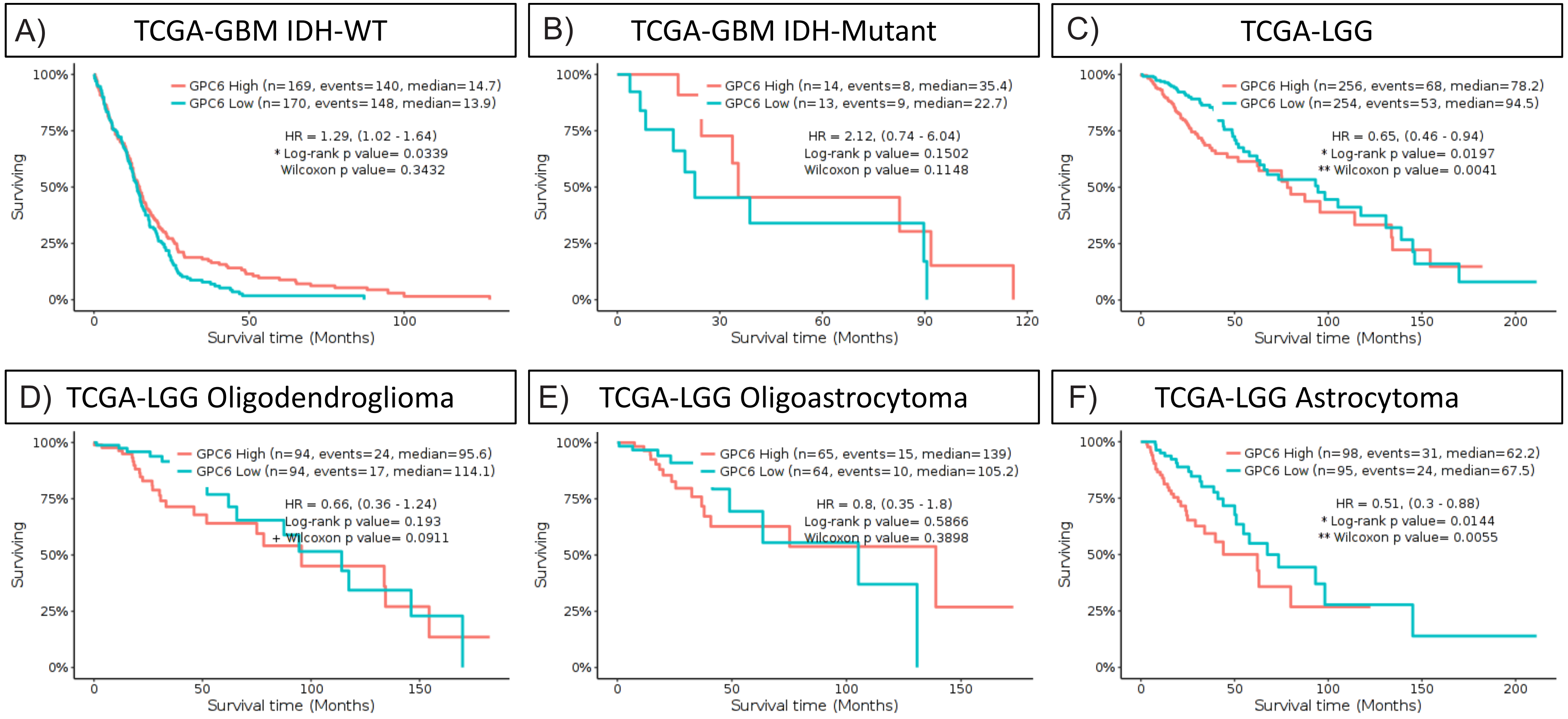

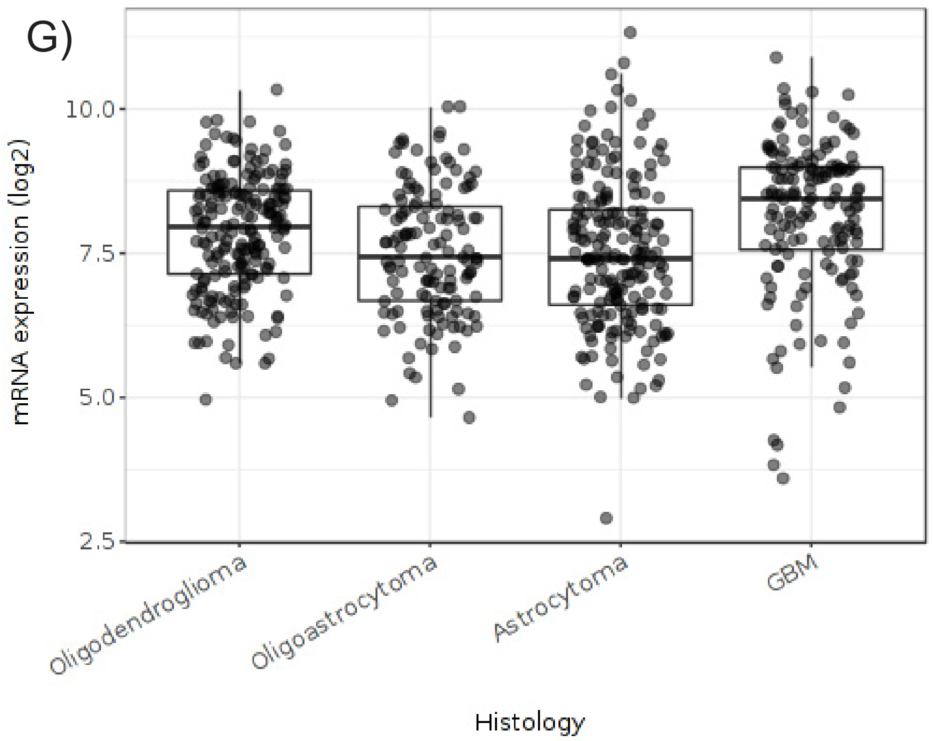

H) Comparison	Tukey's p.adj	Pairwise t-test
GBM-Astrocytoma	0.00	5.5E-06
GBM-Oligoastrocytoma	0.00	7.8E-05
GBM-Oligodendroglioma	0.11	1.4E-01
Astrocytoma-Oligoastrocytoma	1.00	1.0E+00
Oligoastrocytoma-Oligodendroglioma	0.07	9.0E-02
Astrocytoma-Oligodendroglioma	0.02	2.8E-02

Supplementary Fig. 5 (supplement to Figure 2): survival as a function of GPC6 expression

A-F) Kaplan-Meier survival curves differentiating across different glioma subtypes. Data was derived from TCGA. Curves were generated from the GlioVis tool (REF: Bowman et al, Neuro-Oncol 2017, PMID: 28031383). Each curve compares the survival of the upper and lower halves of GPC6 expression within patient tumors. G) Box-whisker plot differentiating GPC6 expression across different glioma subtypes. All box-whisker plots center on the median; the bounds of the boxes mark the upper and lower quartile; the whiskers extend to the upper and lower extremes (1.5x interquartile range from the upper and lower quartiles). H) p-values when comparing the expression data (from G) across different glioma subtypes using Tukey's and Pairwise t-test.

Supplementary figure 6 (supplement to figure 3)

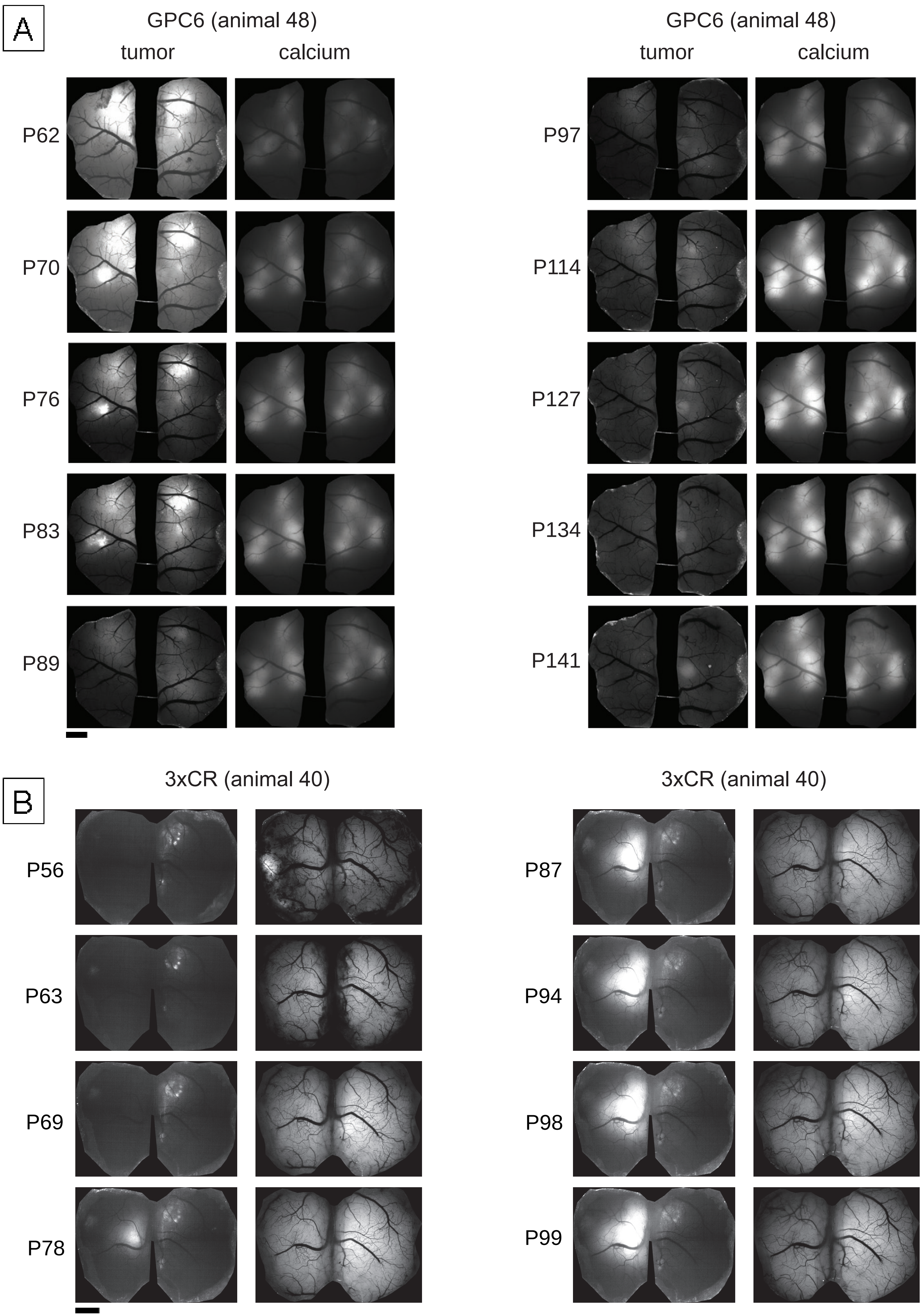

Supplementary Fig. 6 (supplement to Figure 3): Long-term stability of 1-photon widefield fluorescence.

A) Widefield, 1-photon fluorescence images from a GPC6 tumor animal, taken across 10 time points between P62 and P141 (11 weeks). In each time point, the left image shows tumor fluorescence (tumor labeled with GFP, green emission filter, red fluorescence excitation), and the right image show a calcium indicator (AAV-jrGeCO1a, red emission, green excitation light). Scale bar = 1 mm. Note optical clarity remains high throughout the experiment as seen by visibility of small blood vessels. Overall brightness dynamics were normalized by background signal detected inside the lumen of the large pial vessels, whose brightness is independent of the tumor or calcium signal. Similar results were obtained in 5 other animals over comparable time spans.

B) Analogously to A), here we show an example of a 3xCR tumor mouse that was imaged over 7 weeks (P56-99) with a different combination of fluorophores: The tumor was labeled with BFP (blue fluorescence emission, 400nm excitation), and calcium visualized with a genetically expressed indicator (thy1-GCaMP6s, green light emission, blue light excitation). As in the GPC6 example in A), there was no significant loss of optical clarity over time. Similar results were obtained in 10 other animals over comparable time spans.

Supplementary figure 7 (supplement to figure 4)

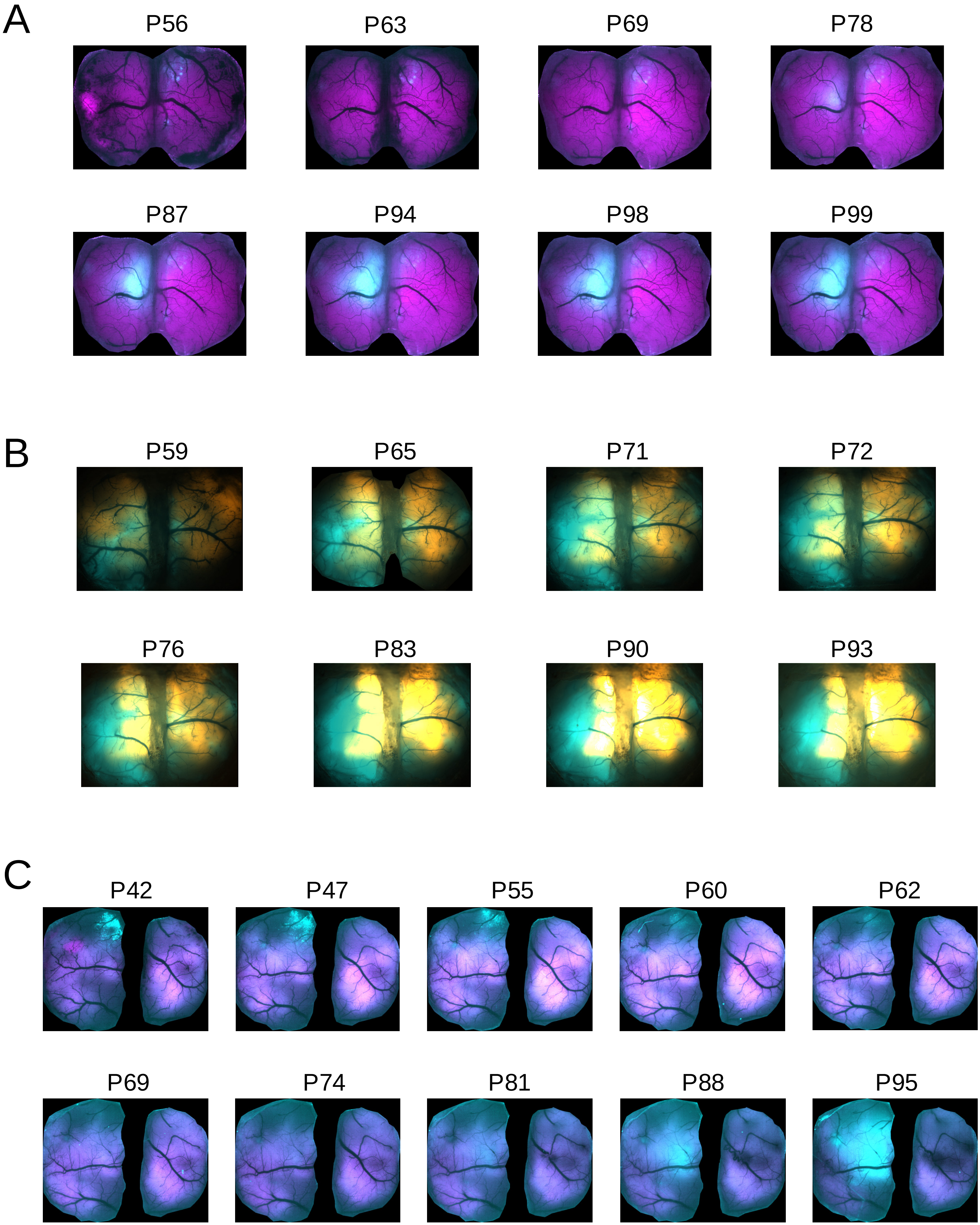

Supplementary Fig. 7 (supplement to Figure 4): Dual-channel overlay images showing calcium/glutamate and tumor fluorescence

A) Widefield, 1-photon fluorescence overlay images from a 3xCR tumor animal, taken across 8 time points between P56 and 99, tumor = cyan, GCaMP6s = magenta. Similar results were obtained in 5 other comparable animals.
B) Widefield overlay images from a 3xCR tumor animal, taken across 8 time points between P59 and P93, tumor = cyan, iGluSnfr = orange/yellow. Similar results were obtained in 4 other comparable animals.
C) Widefield overlay images from a GPC6 tumor animal, taken across 10 time points between P42 and P95, tumor = cyan, GCaMP8m = magenta. Similar results were obtained in 5 other comparable animals.

Supplementary figure 8 (supplement to figure 5)

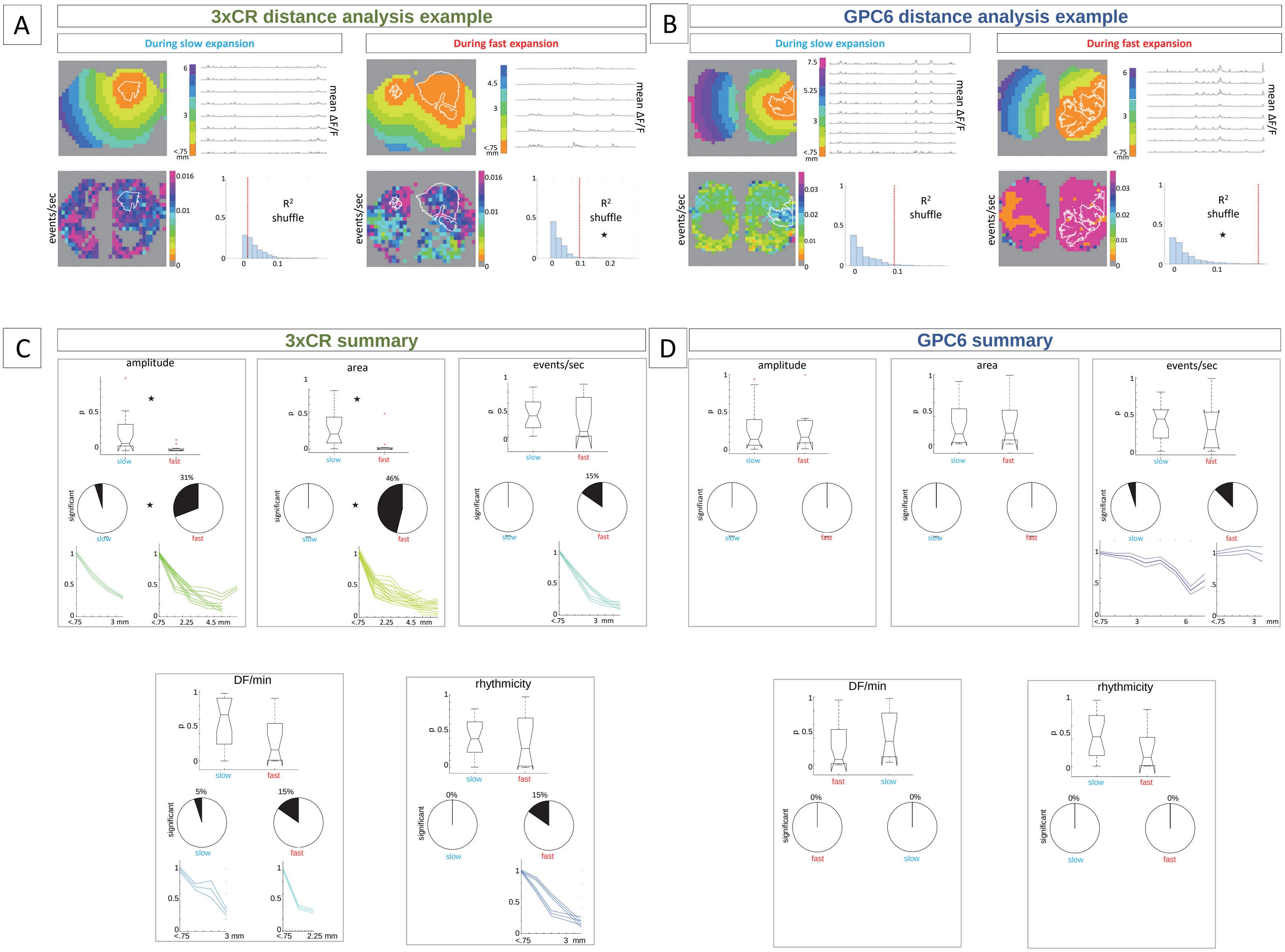

Supplementary Fig. 8 (Supplement to Figure 5): Aggregate neural activity patterns are significantly elevated when the tumor growth rate exceeds $10^5 \mu m^2$/day

A) Example of two 3xCR tumor recordings in the same animal at a time point of slow tumor growth rate (4 left panels) and fast growth rate (4 right panels). Top left panel: The distance from the tumor edge was computed for each pixel in the spatially downsampled calcium movies. Distance bands are color-coded in 0.75 mm increments. Top right: The mean ΔF/F traces to the right of each distance-band plot depict a 30-sec period of mean quiet spontaneous activity corresponding to the adjacent color scale. Bottom left: To illustrate the approach, each pixel was color-coded according to the mean event rate across the analyzed recording time. Bottom left: neuronal event rates were fitted against distance from the tumor to generate a goodness-of-fit R^2 value. Pixel distances were circularly shuffled 500k times to generate a null distribution of the linear fit R^2 (blue histogram) and to compute the corresponding p-value of the fit significance. (red line in the histogram of the shuffled null distribution, $p = 0.62$). In the example from a fast-growth epoch (same animal) at the right, event rates were significantly higher close to the tumor than farther away ($p = 0.02$)).

B) As in A, here we show example data from a GPC6 tumor animal from a recording during slow tumor growth (4 left panels), and one while growing fast (4 right panels): color-coded distance bands around the tumor, 30 seconds of spontaneous activity corresponding to those distances, color-coded event rate levels for all pixels, and significance of linear goodness-of-fit R^2 with shuffled null distributions.

C) 3xCR tumor animal recordings with significant relationships between distance and activity pattern metrics were identified using the method shown in A and B. Data from $n = 6$ animals, 20 recordings under slow tumor expansion, 13 during fast expansion.

D) GPC6 recordings ($n=4$ animals) were analyzed as in C. Data are from 20 recordings under slow growth conditions and 8 recordings during fast growth. Note the lack of significant relationships between distance and activity metrics in contrast with the 3xCR recordings. Box plots in C and D center around the median. Non-overlapping notches around the median correspond to groups having different medians at the 95% significance level. The upper and lower bounds of the box are the upper and lower quartile, the whiskers represent non-outlier minimum/maximum.

Supplementary figure 9 (supplement to figure 6)

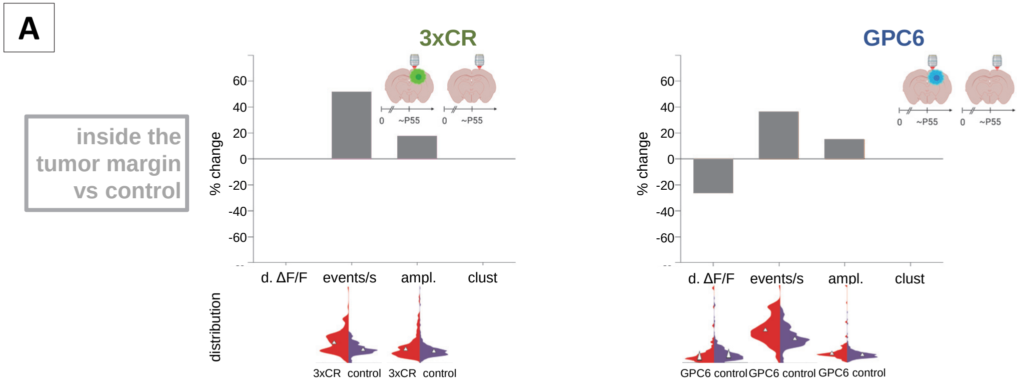

Supplementary Fig. 9 (supplement to figure 6): neural activity patterns inside both 3xCR and GPC6 tumor margins are qualitatively different than in tumor-free control animals.

A) The left panel shows the comparison between neuronal activity parameters inside 3xCR tumor margins versus non-tumor control animals, and the right panel compares activity inside GPC6 tumor margins with controls. The bar graphs were computed by pooling data from several animals in which comparisons were already shown to be statistically significant. Therefore they do not have error bars and are showing the percent change value for each metric directly.

Supplementary figure 10

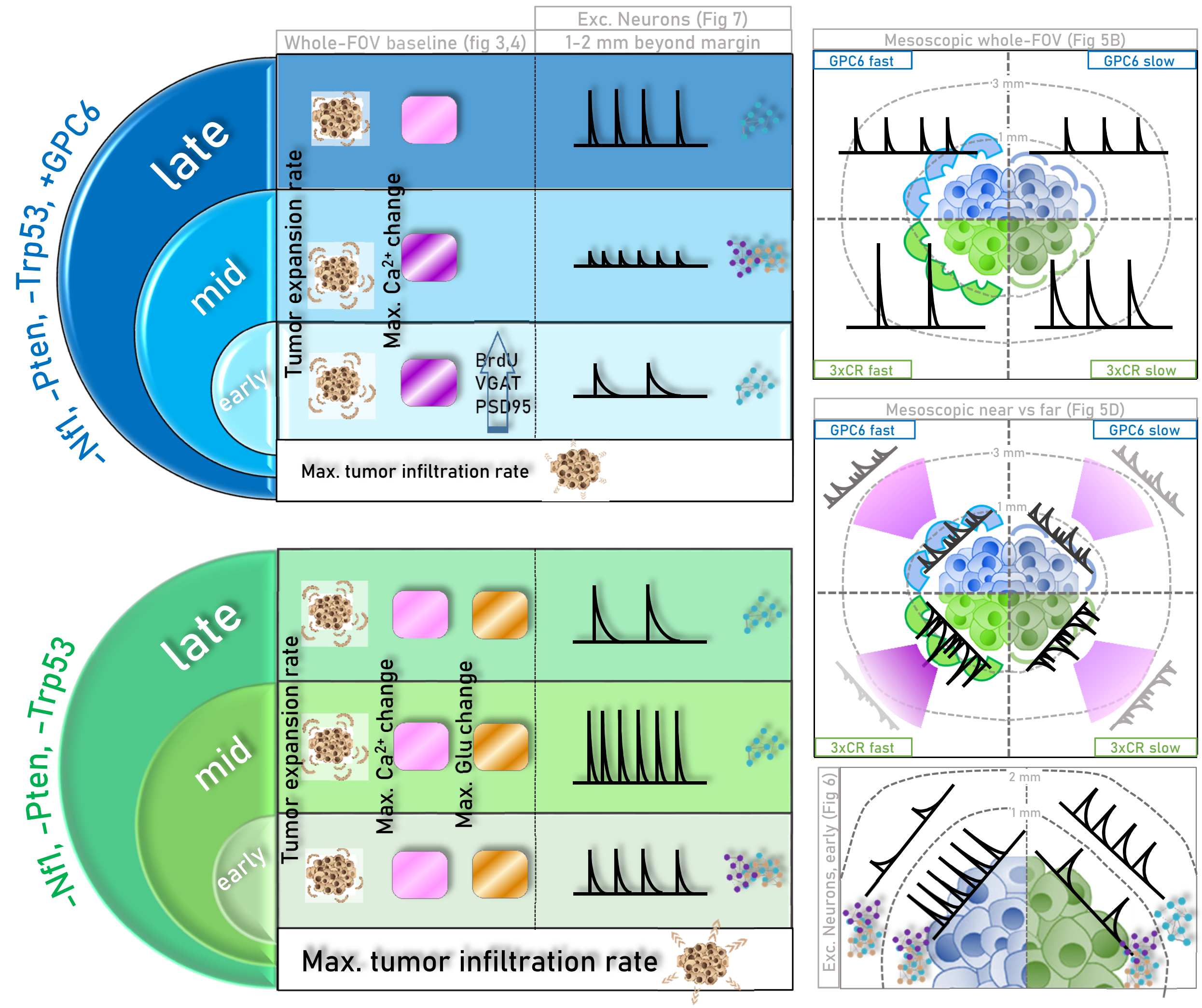

Supplementary Fig. 10: Schematic cartoon summary of the main findings in this study.

This schematic cartoon summarizes the main findings of this study in one figure. Each section summarizes results from one or two figures (grey headings). Black activity traces can represent either cellular (dΔF/F) or mesoscopic (ΔF/F) activity.

Supplementary table 1

tumor genotype	animal #	sex m/f	reporter	promoter	recorded time span (day P)	data used for figures
3xCR	10	m	AAV-jrGeco	hsyn	54-82	5
3xCR	15	f	AAV-G7f	hsyn	58-107	4,5
3xCR	18	m	AAV-G7f	hsyn	59-94	4
3xCR	19	m	AAV-iGluSnFr	EF1α	64-80	4
3xCR	20	m	AAV-iGluSnFr	EF1α	69-83	4
3xCR	21	m	AAV-G7f	hsyn	63-129	4,5
3xCR	22	f	AAV-iGluSnFr	EF1α	59-93	4
3xCR	23	f	AAV-G7f	hsyn	50-99	4
3xCR	28	f	G6s	thy1	48-125	4,5,7
3xCR	29	f	G6s	thy1	51-83	4
3xCR	34	m	G6s	thy1	63-100	4
3xCR	37	m	G6s	thy1	43-127	7
3xCR	38	m	G6s	thy1	49-111	4,5,6,7
3xCR	40	f	G6s	thy1	56-99	4,5,6,7
3xCR	68	f	AAV-iGluSnFr	CaMKIIα	46-93	4
3xCR	79	m	AAV-iGluSnFr	CaMKIIα	53-90	4
3xCR	84	m	AAV-G8m	CaMKIIα	50-64	7
3xCR	85	m	AAV-G8m	CaMKIIα	43-67	6,7
3xCR	86	m	AAV-G8m	CaMKIIα	46-71	4,6,7
GPC6	48	f	AAV-jrGeco	hsyn	62-141	4,5
GPC6	49	f	AAV-jrGeco	hsyn	61-96	4,5
GPC6	51	f	AAV-jrGeco	hsyn	62-195	4
GPC6	70	m	AAV-G8m	CaMKIIα	42-70	4,6,7
GPC6	71	f	AAV-G8m	CaMKIIα	40-69	4,5,6,7
GPC6	72	m	AAV-G8m	CaMKIIα	42-106	4,5,6,7
GPC6	73	f	AAV-G8m	CaMKIIα	41-102	4,5,6,7
GPC6	74	m	AAV-G8m	CaMKIIα	35-47	6,7
GPC6	75	m	AAV-G8m	CaMKIIα	42-60	4,6,7

Supplementary Table 1:

Here we list all animals used to collect imaging data for this study. We specify tumor genotype, internal animal ID, sex, fluorescent activity reporter type, promoter, postnatal day of first and last recording, and the figure numbers in which data from each animal were included.
