# Supplementary material for: Glioblastoma disrupts cortical network activity at multiple spatial and temporal scales
Source: Nat Commun. 2024 May 27;15:4503. doi: 10.1038/s41467-024-48757-5 (PMC11130179; doi:10.1038/s41467-024-48757-5)
Supplement: Supplementary file 3 — Reporting Summary [file 41467_2024_48757_MOESM3_ESM.pdf]

nature portfolio

Corresponding author(s): Jeffrey Noebels, Jochen Meyer

Last updated by author(s): Apr 12, 2024

Reporting Summary

Nature Portfolio wishes to improve the reproducibility of the work that we publish. This form provides structure for consistency and transparency in reporting. For further information on Nature Portfolio policies, see our Editorial Policies and the Editorial Policy Checklist.

Statistics

For all statistical analyses, confirm that the following items are present in the figure legend, table legend, main text, or Methods section.

n/a	Confirmed	
☐	☒	The exact sample size (n) for each experimental group/condition, given as a discrete number and unit of measurement
☐	☒	A statement on whether measurements were taken from distinct samples or whether the same sample was measured repeatedly
☐	☒	The statistical test(s) used AND whether they are one- or two-sided<br>*Only common tests should be described solely by name; describe more complex techniques in the Methods section.*
☐	☒	A description of all covariates tested
☐	☒	A description of any assumptions or corrections, such as tests of normality and adjustment for multiple comparisons
☐	☒	A full description of the statistical parameters including central tendency (e.g. means) or other basic estimates (e.g. regression coefficient) AND variation (e.g. standard deviation) or associated estimates of uncertainty (e.g. confidence intervals)
☐	☒	For null hypothesis testing, the test statistic (e.g. F, t, r) with confidence intervals, effect sizes, degrees of freedom and P value noted<br>*Give P values as exact values whenever suitable.*
☒	☐	For Bayesian analysis, information on the choice of priors and Markov chain Monte Carlo settings
☐	☒	For hierarchical and complex designs, identification of the appropriate level for tests and full reporting of outcomes
☒	☐	Estimates of effect sizes (e.g. Cohen's d, Pearson's r), indicating how they were calculated

Our web collection on statistics for biologists contains articles on many of the points above.

Software and code

Policy information about availability of computer code

Data collection	Bruker Prairieview 5.63, Matlab 202a/2021a, WinEDR v3.8.7, pco camware 4.13
Data analysis	Matlab 2021a/2023a, R 4.3.1, GEPIA2. CUstom Matlab data analysis routines are available on Github/Zenodo (DOI 10.5281/zenodo.10954325)

For manuscripts utilizing custom algorithms or software that are central to the research but not yet described in published literature, software must be made available to editors and reviewers. We strongly encourage code deposition in a community repository (e.g. GitHub). See the Nature Portfolio guidelines for submitting code & software for further information.

Data

Policy information about availability of data

All manuscripts must include a data availability statement. This statement should provide the following information, where applicable:

- Accession codes, unique identifiers, or web links for publicly available datasets
- A description of any restrictions on data availability
- For clinical datasets or third party data, please ensure that the statement adheres to our policy

Data for suppl. fig 4: Single cell RNA-Seq analysis of adult and pediatric GBM from publicly available dataset (REF: Neftel et al, Cell 2019, PMID: 31327527); spatial transcriptomic data from publically available dataset (REF: Puchalski et al, Science 2018, PMID: 29748285);
Data for suppl. fig 5: generated from the GlioVis tool (REF: Bowman et al, Neuro-Oncol 2017, PMID: 28031383).
The RNAseq data (figure 2) is available at https://www.ncbi.nlm.nih.gov/geo/query/acc.cgi?acc=GSE263832

The raw imaging data that support the findings of this study are available under restricted access for reasons of sensitivity, access can be obtained by contacting the lead author (jfmeyer@bcm.edu) within 2 weeks upon request. Data are located in controlled access data storage at Baylor College of Medicine. Source data of all graphs in figures are provided with this paper.

Research involving human participants, their data, or biological material

Policy information about studies with human participants or human data. See also policy information about sex, gender (identity/presentation), and sexual orientation and race, ethnicity and racism.

Reporting on sex and gender	Human glioma tissue microarrays were provided by Baylor College of Medicine's Pathology and Histology Core without sex and gender information.
Reporting on race, ethnicity, or other socially relevant groupings	Human glioma tissue microarrays were provided by Baylor College of Medicine's Pathology and Histology Core without race/ethnicity information.
Population characteristics	Human glioma tissue microarrays were provided by Baylor College of Medicine's Pathology and Histology Core without population information.
Recruitment	Human glioma tissue microarrays were provided by Baylor College of Medicine's Pathology and Histology Core without recruitment information.
Ethics oversight	Baylor College of Medicine

Note that full information on the approval of the study protocol must also be provided in the manuscript.

Field-specific reporting

Please select the one below that is the best fit for your research. If you are not sure, read the appropriate sections before making your selection.

☒ Life sciences ☐ Behavioural & social sciences ☐ Ecological, evolutionary & environmental sciences

For a reference copy of the document with all sections, see nature.com/documents/nr-reporting-summary-flat.pdf

Life sciences study design

All studies must disclose on these points even when the disclosure is negative.

Sample size	Sample sizes were based upon previously published variability of assays and sufficient numbers for duplication: Using the same GBM mouse model, 2 previous studies showed significant changes in EEG spiking upon tumor invasion over time; group sizes of 3-5 animals were deemed sufficient (Yu et al, Nature 2020, PMID 31996845; Hatcher et al, JCI 2020, PMID 32250339). Here, each group of animals included at least 4 animals for the 2-photon imaging data, and 5 animals for the widefield imaging data.
Data exclusions	Chronic imaging data were excluded from animals that died before reaching the time points required for comparative longitudinal analysis of tumor expansion rates and peritumoral neural activity.
Replication	To ensure results were reproducible, data from multiple independent IUE cohorts of 3xCR animals (2 cohorts for 2-photon imaging, 3 cohorts for widefield imaging), and GPC6 tumor animals (2 cohorts for all imaging data) were included for statistical comparisons. Animals with the same tumor genotype showed similar results regardless of which cohort they originated from. The detailed table in the supplementary material lists numbers of animals were used to replicate results for each figure.
Randomization	Each IUE cohort consisted of ~50% more animals than were recorded from, and experimental mice were chosen randomly. For analysis of neuronal activity, neurons from larger FOVs were chosen randomly to assure each animal was represented by the same number of cells.
Blinding	Analysis of human transcriptomics, survival, ISH, gene ontology data: Blinding of the authors does not apply as the underlying source data originated either from publicly available datasets or images provided by the Baylor College of Medicine Pathology and Histology Core. Mouse survival, IHC, and RNAseq data: Blinding was not feasible due to the nature of the multi-step experimental protocols that did not allow for de-identified tracking of samples. Imaging data: For mouse in vivo data acquisition, blinding was not possible because longitudinal imaging experiments using the two tumor genotypes were run in separate cohorts due to logistical constraints concerning both acquisition and analysis, however data were universally analyzed in an unbiased way.

Reporting for specific materials, systems and methods

We require information from authors about some types of materials, experimental systems and methods used in many studies. Here, indicate whether each material, system or method listed is relevant to your study. If you are not sure if a list item applies to your research, read the appropriate section before selecting a response.

Materials & experimental systems

n/a	Involved in the study
☐	☒ Antibodies
☒	☐ Eukaryotic cell lines
☒	☐ Palaeontology and archaeology
☐	☒ Animals and other organisms
☒	☐ Clinical data
☒	☐ Dual use research of concern
☒	☐ Plants

Methods

n/a	Involved in the study
☒	☐ ChIP-seq
☒	☐ Flow cytometry
☒	☐ MRI-based neuroimaging

Antibodies

Antibodies used

rat anti-BrdU (BUl/75 (ICRl), 1:200; abeam, en: ab6326, In : GR3269246-l), mouse anti-gephyrin (1:500; Synaptic Systems, en: 147011, In : 1-64), rabbit anti-GFP (1 :1,000; ThermoFisher, A-11122), mouse anti-PSD95 (7E3-1B8, 1:500; ThermoFisher, en : MAl-046, In: Lll47875), guinea-pig anti-VGAT (1 :500; Synaptic Systems, en: 131004, In: 2-41), guinea-pig anti-VGLUTl (1:2,000; Millipore, en : AB5905, In: 3193844). We used species-specific secondary antibodies tagged with Alexa Fluor 488,568, or 647 (1 :1,000, ThermoFisher) for immunofluorescence.

Validation

The following URLs are the validation statements provided by each manufacturer/distributor of the antibodies used in this study. In addition, included are the product pages of each of the antibodies which include a list of references where the product was used in a peer-reviewed study.
ABCAM: https://www.abcam.com/primary-antibodies/how-we-validate-our-antibodies
Synaptic Systems: https://sysy.com/resources/antibody-validation
ThermoFisher: https://www.thermofisher.com/us/en/home/life-science/antibodies/invitrogen-antibody-validation.html
Millipore: https://www.emdmillipore.com/US/en/life-science-research/antibodies-assays/antibodies-overview/Antibody-Development-and-Validation/cFOb.qB.8McAAAFOb64qQvSS,nav

rat anti-BrdU (BUl/75 (ICRl), 1:200; abeam, cn: ab6326, In: GR3269246-l),
https://www.abcam.com/products/primary-antibodies/brdu-antibody-bu175-icr1-proliferation-marker-ab6326.html

mouse anti-gephyrin (1:500; Synaptic Systems, cn: 147011, In: 1-64),
https://sysy.com/product/147011

rabbit anti-GFP (1 :1,000; ThermoFisher, A-11122),
https://www.thermofisher.com/antibody/product/GFP-Antibody-Polyclonal/A-11122

mouse anti-PSD95 (7E3-1B8, 1:500; ThermoFisher, cn :MA1-046, In: Lll47875),
https://www.thermofisher.com/antibody/product/PSD-95-Antibody-clone-7E3-1B8-Monoclonal/MA1-046

guinea-pig anti-VGAT (1 :500; Synaptic Systems, cn: 131004, In: 2-41),
https://sysy.com/product/131004

guinea-pig anti-VGLUT1 (1:2,000; Millipore, cn: AB5905, In: 3193844).
https://www.sigmaaldrich.com/US/en/product/mm/ab5905

Animals and other research organisms

Policy information about studies involving animals; ARRIVE guidelines recommended for reporting animal research, and Sex and Gender in Research

Laboratory animals

CD-1 and C57-BL6 background mice were housed at 21 deg Celsius under a 12h/12h on/off light cycle. Humidity in the vivarium was set to 45%. See supplementary table 1 for precise age ranges during recording for each animal used in the imaging study.

Wild animals

The study did not involve wild animals.

Reporting on sex

Animals of both sexes were used in the study; sex was not a factor in experimental design and was not considered in random animal assignments to experimental groups.

Field-collected samples

The study did not involve samples collected from the field.

Ethics oversight

Baylor College of Medicine IACUC

Note that full information on the approval of the study protocol must also be provided in the manuscript.

Plants

Seed stocks	*Report on the source of all seed stocks or other plant material used. If applicable, state the seed stock centre and catalogue number. If plant specimens were collected from the field, describe the collection location, date and sampling procedures.*
Novel plant genotypes	*Describe the methods by which all novel plant genotypes were produced. This includes those generated by transgenic approaches, gene editing, chemical/radiation-based mutagenesis and hybridization. For transgenic lines, describe the transformation method, the number of independent lines analyzed and the generation upon which experiments were performed. For gene-edited lines, describe the editor used, the endogenous sequence targeted for editing, the targeting guide RNA sequence (if applicable) and how the editor was applied.*
Authentication	*Describe any authentication procedures for each seed stock used or novel genotype generated. Describe any experiments used to assess the effect of a mutation and, where applicable, how potential secondary effects (e.g. second site T-DNA insertions, mosiacism, off-target gene editing) were examined.*
